# Potential Anticancer Properties and Mechanisms of Action of Formononetin

**DOI:** 10.1155/2019/5854315

**Published:** 2019-07-28

**Authors:** Dongjun Jiang, Azhar Rasul, Rabia Batool, Iqra Sarfraz, Ghulam Hussain, Muhammad Mateen Tahir, Tian Qin, Zeliha Selamoglu, Muhammad Ali, Jiang Li, Xiaomeng Li

**Affiliations:** ^1^The Key Laboratory of Molecular Epigenetics of MOE, Institute of Genetics and Cytology, Northeast Normal University, Changchun 130024, China; ^2^Department of Zoology, Faculty of Life Sciences, Government College University Faisalabad (GCUF), 38000, Pakistan; ^3^Department of Physiology, Faculty of Life Sciences, Government College University Faisalabad (GCUF), 38000, Pakistan; ^4^Department of Medical Biology, Faculty of Medicine, Nigde Ömer Halisdemir University, Nigde, Campus 51240, Turkey; ^5^Quaid-e-Azam University, Islamabad 45320, Pakistan; ^6^Dental Hospital, Jilin University, Changchun 130021, China

## Abstract

Nature, a vast reservoir of pharmacologically active molecules, has been most promising source of drug leads for the cure of various pathological conditions. Formononetin is one of the bioactive isoflavones isolated from different plants mainly from* Trifolium pratense, Glycine max, Sophora flavescens, Pycnanthus angolensis, *and* Astragalus membranaceus*. Formononetin has been well-documented for its anti-inflammatory, anticancer, and antioxidant properties. Recently anticancer activity of formononetin is widely studied. This review aims to highlight the pharmacological potential of formononetin, thus providing an insight of its status in cancer therapeutics. Formononetin fights progression of cancer via inducing apoptosis, arresting cell cycle, and halting metastasis via targeting various pathways which are generally modulated in several cancers. Although reported data acclaims various biological properties of formononetin, further experimentation on mechanism of its action, medicinal chemistry studies, and preclinical investigations are surely needed to figure out full array of its pharmacological and biological potential.

## 1. Introduction

Natural products have served as an infinite reservoir of various diversified chemical compounds, driving pharmaceutical industries for many years [1]. In the discipline of cancer therapeutics, natural products hold a great potential. It has been reported that, from 1981 to 2014, about 1562 drugs were approved out of which 1211 drugs were derived from small molecules that are nonsynthetic [2]. About 50% of the medicines and 48.6% of anticancer drugs are derivatives of natural products [3]. Chinese encyclopedia that dates back to 2000 BC has reported that 1898 herbal products have been used as medicines [4]. In accordance to World Health Organization, about 80% of population depends upon plant-derived traditional medicines. This drug discovery approach exhibits far-reaching domain where large-scale research could provide novel leads against cellular or molecular targets [5].

Different pharmacological studies on natural products have proclaimed their authenticity as potent anticancer [6], antioxidant [7], and anti-inflammatory agents [8].

Triterpenoid saponins and isoflavones belong to family of amphiphilic glycosides which are naturally present in medicinal plants, herbs, and marine organisms. Saponins and isoflavones have major role in folk medicine due to their biological and pharmacological properties [9]. These secondary metabolites possess various anticancer, anti-inflammatory, and antioxidant properties. Epidemiological studies recommend that intake of food enriched with isoflavonoids reduces the risk of various cancers [9].

Formononetin, an isoflavone isolated from soy bean and red clover, has been known to be endowed with numerous pharmacological attributes such as anticancer [10], anti-inflammatory, [11], and antioxidant attributes [12].

To date, there is no review on the biological potential of formononetin. This article intends to focus on the researches relevant to the biological and pharmacological activities of Formononetin. The literature is searched via different e-sites like PubMed, Elsevier Science Direct, Springer Link, and related journals. Key words which are used for searching are “Formononetin”, “Formononetin and its biological activities”, “anticancer”, and “natural products”.

## 2. Natural Sources of Formononetin

**Figure 1:**
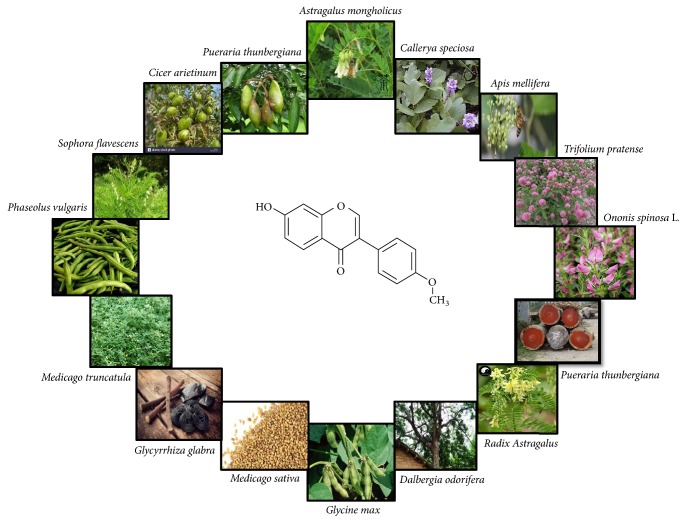
Natural sources of Formononetin.

Formononetin (Figure 1) has been reported to be isolated from different plants of bean family “Fabaceae” which is the 3^rd^ largest family of land plants. Genus* Trifolium *(Fabaceae) contains 250 species, most of which have been documented as rich source of formononetin [13]. In addition to this family, formononetin is also present in different plants including* Trifolium pratense* [14]*, Glycine max* [15, 16],* Sophora flavescens* [17],* Pycnanthus angolensis* [18],* Spatholobus suberectus* [19],* Cicer arietinum* [20],* Dalbergia odorifera, Pueraria thunbergiana* [21],* Actaea racemosa* [22],* Ononis spinosa *L. [9],* Dalbergia ecastaphyllum *[23],* Callerya speciosa* [24],* Astragalus membranaceus* [25], and* Astragalus mongholicus* as shown in Figure 1. The list of plants having Formononetin, their parts, and pharmacological properties are elaborated in Table 1.

## 3. Biological Activities of Formononetin and Mechanisms of Its Action

**Figure 2:**
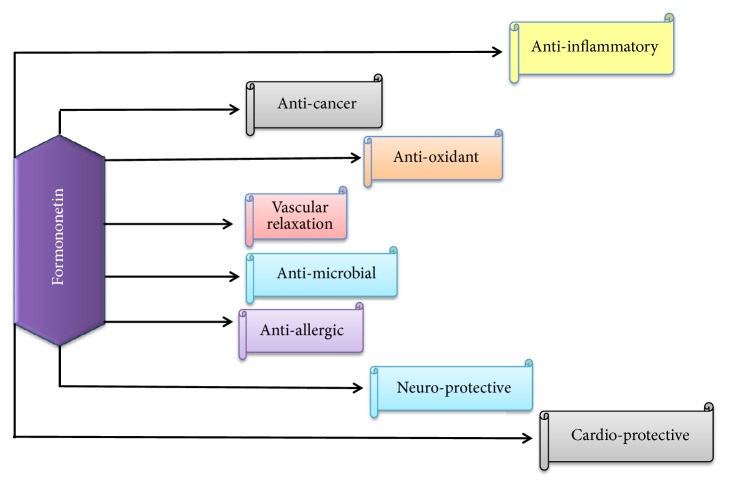
Biological activities of Formononetin.

The biological and pharmacological activities of bioactive compound “Formononetin” are well-documented as antitumor activity, [23] antiproliferative activity [14], growth inhibitory activity [55], vasorelaxant action [56], neuroprotective effect [57], antiapoptotic activity [48], cardioprotective activity [58], mammary gland proliferative activity [59], antioxidant activity [60], antimicrobial activity, and anti-inflammatory activity [15] as provided in Figure 2. Numerous* in vivo/in vitro* investigations have been done on Formononetin to uncover its biological attributes and mechanisms of actions.

### 3.1. Anticancer Activity

Cancer is uncontrolled proliferation of cells which occurs due to genetic or epigenetic modifications and signaling defects in cells [61]. Different synthetic drugs are used for the treatment of this deadly disease [2]. Due to the limited success of synthetic drugs, it is necessary to identify novel natural products having the ability to induce apoptotic cell death and arrest cell cycle in tumor cells without toxic effect on normal cells [62]. Various studies have declared that formononetin can block, delay, or inhibit the initiation and progression of cancer. This review intends to focus on the studies relevant to anticancer potential of Formononetin which will allow the researchers to further investigate this novel chemical entity as a potential anticancer drug candidate.

**Figure 3:**
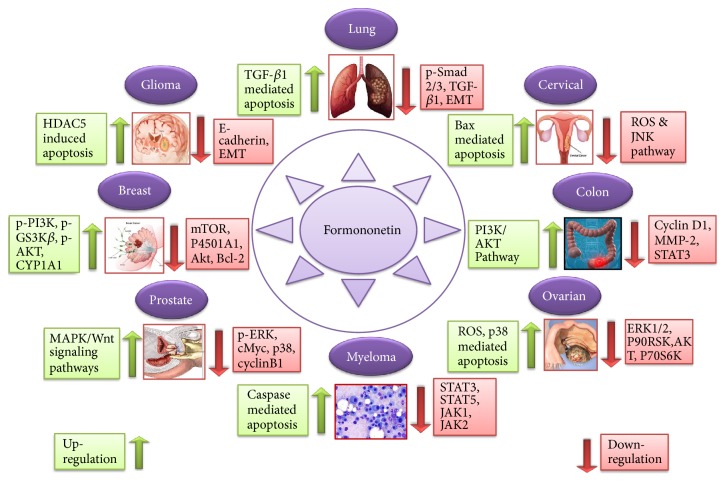
Formononetin cytotoxic effects against various cancer types via different signaling pathways.

Currently, it is documented that about 60% drugs that are used for cure of cancer are derived from natural sources [63]. Secondary metabolites isolated from natural products encompassing terpenes, alkaloids, isoflavones, and polyphenols have been reported as anticancer agents [64, 65]. Anticancer properties of formononetin have been documented against several types of cancer [66] such as breast [67], colon [41], glioma [54], osteosarcoma [68], multiple myeloma [52], adrenal medulla [51], nasopharyngeal [50], prostate [49], bladder [45], laryngeal [23], lung [43], and cervical cancer [42] (Figure 3).

#### 3.1.1. Formononetin and Cell Cycle Arrest

Uncontrolled divisions of cells are key trait of cancer cells [69]. Natural compounds have ability to prohibit the functions of cyclin dependent kinases and various other cell cycle regulatory proteins, thus causing cell cycle arrest [70].

**Figure 4:**
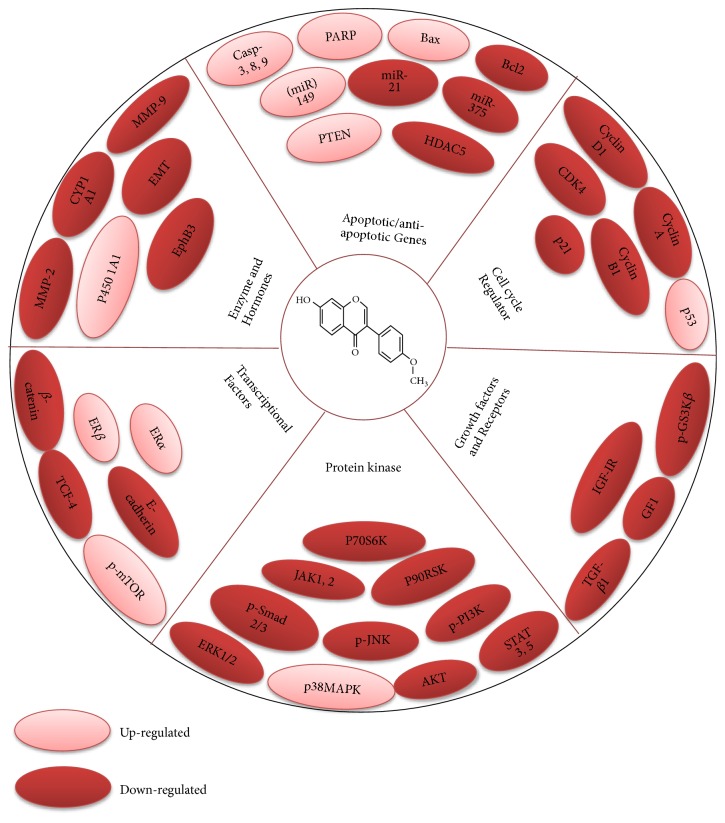
Diagram presenting mechanism of action and molecular targets for Formononetin resulting in chemotherapeutic activity.

Formononetin has been documented to arrest the cell cycle at various stages [44]. In human ovarian cancer, Formononetin leads cancer cells towards apoptosis and arrested cell cycle at G0/G1 phase in ES2 and OV90 cell lines [10]. Formononetin downregulated cyclin D1 which further arrested the cell cycle at the phase of G0/G1 in HCT-116 cells [41]. Formononetin arrested cells at G0/G1 phase in HepG2 cancerous cells [23]. In lung cancer cells, it caused the arresting of cell cycle at G1 phase via alteration of the p21, cyclin A, and cyclin D expression [44]. In PC-3 and DU145 cells, this compound has the ability to arrest the cells at G0/G1 phase via reducing AKT/cyclin D1/CDK4 expression [49] (Figure 4).

It can be summarized that Formononetin causes the arrest the cells at G0/G1 phase but it needs to be investigated whether it has arrested the cellular cycle at G0 or G1 phase. Furthermore, it is interesting for researchers to investigate whether Formononetin could arrest the cells at G2/M or S-phase. Thus, further mechanistic investigations are yet obligatory to understand the mechanisms by which Formononetin arrests the cell cycle in various cancers.

#### 3.1.2. Formononetin and Apoptosis

Apoptosis is a systematic and synchronized way of cell death which is peculiarized by different morphological features including formation of blebs on cell membrane, condensation of chromosomes, and fragmentation of nucleus [71, 72]. It is reported that certain cellular signals are necessary to regulate the growth of cells but mutation in these signals prevent the cells to undergo apoptosis [73, 74]. Accumulated data indicated that various chemopreventive agents have been identified that can lead cancer cells towards apoptosis [75, 76]. Formononetin has the ability to inhibit or block the growth of cancerous cells via intrinsic or extrinsic apoptosis pathways [49].

Formononetin has emerged as novel agent for its chemotherapeutic activity [52]. Anticancer activity of Formononetin has been known to be associated with induction of apoptosis via activation of caspase family, ROS, activation of Bax, downmodulation of antiapoptotic protein Bcl-2, [53], upregulation of p-AKT [37], inhibiting the activation of NF-*κ*B, reduction of ERK1/2 [10], inhibition of MMP-2/-9, upregulation of p38, p21, and p53 [10, 44], increase of the level of P450 1A1 [36], and blockage of PI3K/AKT, STAT3 signaling pathways [41] (Table 2) (Figure 4).

(*1) Formononetin and MAPK and PI3K/AKT Pathway.* MAPK, mitogen-activated protein kinase pathway, performs significant function in cell division, differentiation, proliferation, and cellular apoptosis [77]. MAPK pathway has four distinct signaling domains such as ERK, JNK, BMK-1, and p38 MAPK. Extracellular signals conducted by these kinases regulate the process of cellular apoptosis and growth [78, 79]. MAPK pathway has been reported as novel target to combat cancer. The PI3K/AKT pathway also performs a significant role in tumor development. This activated pathway is affiliated with several cancer types. Therefore, targeting this pathway has the ability to combat cancer [80].

Isoflavonoids have been documented as anticancer agents that inhibit cancer cell proliferation and have antimetastatic effects [35, 81]. Formononetin has ability for the induction of apoptosis in breast cancerous cells via activating Ras/p38 MAPK pathway in a dose dependent mode [40].

Formononetin reduced the p-AKT, p-P90RSK, p-S6, p-ERK1/2, and p-P70S6K proteins as well as enhancing the levels of p-p38 MAPK to mediate cellular proliferation and apoptosis in OV90 and ES2 cells [10]. Treatment with formononetin enhanced the levels of ER*α* and p-AKT in HUVEC and MCF-7 cancer cells [37]. This natural compound Formononetin suppresses proliferation and invasive capability of cells by inhibiting MMP-2/-9 via inactivation of phosphorylation of AKT and PI3K in HCT-116 and SW1116 cells [37, 41]. Formononetin also exerts its anticancer effects due to downmodulation of p-AKT, miR-21 expression, and upregulation of PTEN in T24 cells [45]. Formononetin inhibited the proliferation via inactivation of PI3K/ AKT pathway, enhancing the Bax, and downmodulating the Bcl-2 expression [68]. Formononetin and its derivatives such as dithiocarbamate with IC_50_ value of 1.9 *μ*M possess inhibitory potential against PC3 cells [46] (Table 2).

(*2) Formononetin and JAK/STAT Signaling Pathway.* JAK/STAT pathways mediate the transduction of various signals involving cell division, immunity, tumor development, and cell death. Disruption in JAK/STAT signaling pathways may cause several diseases including cancer and immune disorders [82]. Formononetin decreased the activation of STAT5 and STAT3 by suppressing the nuclear translocation of p-STAT5 and p-STAT3 and also inhibited the activation JAK1 and JAK2 in U266 cells. Formononetin also inhibited the IL-6-induced STAT3 activity which ultimately inhibits the cell viability and activates apoptosis [52]. Formononetin suppresses the cellular invasion and proliferation by inhibition of MMP-2/-9 via inactivation of p-STAT3 pathway in colon carcinoma cells [41].

## 4. *In Vivo *Studies and Biosafety Profile

Formononetin with IC_50_ value 2-6 *μ*M has the ability to promote the expression of p-AKT, miR-375, and Bcl-2 in* in vivo* mice model [37]. Treatment with Formononetin suppresses growth of tumor in* in vivo* tumor mouse model at the dose of 60 mg/kg [49]. An* in vivo* investigation demonstrated that Formononetin combined with other compounds reduced allergic inflammation in mice model via downregulating NF-*κ*B activation [83]. Administration of Formononetin reduced the size, volume, and weight of HeLa tumor in* in vivo* mice model induced by injection of HeLa cells in the posterior flanks of mice [84]. As Formononetin is the imperative components of Chinese folk medicines* Radix Astragalus* and Fukeqianjin which are mostly used as antioxidant and anticancer agents, respectively, therefore, it might serve as safe chemotherapeutic drug candidate [85, 86]. Formononetin turns out to be a fascinated bioactive entity as it combines active chemotherapeutic effect with less toxicity in comparison to other isoflavones. However, safe doses and effectiveness of formononetin in targeted therapeutic fields still need to be executed in future.

## 5. Other Biological Functions

Formononetin is extracted from different plants such as* Dalbergia odorifera* in which Formononetin along with other compounds showed antiallergic and anti-inflammatory activities [87]. Formononetin together with other known compounds acts as active inhibitor of EV-A71 infection [88]. Brazilian* Red propolis* extract containing isoflavonoids such as Formononetin exhibits anti-inflammatory potential in a rat model of inflammation [89]. Isoflavones such as Formononetin isolated from soy bean possess antimicrobial and antioxidant activities with IC_50_ values from 10.6 to 22.6 *μ*g/mL [15]. A study indicates that a methoxy Isoflavone, Formononetin, has potential for bone healing process in mouse model and has promising role in fracture-repair process [90]. Hydroalcoholic extract of* Red propolis* containing* Red propolis* has the ability to repair axon after sciatic nerve injury in rat model [91].

## 6. Conclusion and Future Perspectives

In this review article, we have suggested that Formononetin is a good drug candidate with promising pharmacological activities. Various researches have documented the potential applications of Formononetin both* in vivo* and* in vitro*. Being an important bioactive constituent of edible foods such as soybean, chickpeas, and alfalfa beans, Formononetin might turn up as a safe chemotherapeutic drug candidate. Many studies have revealed that formononetin is an inducer of apoptosis in many cancerous cells, but still mechanism of its action is not fully clarified. After the analysis of data, we have found that Formononetin is most active against nasopharyngeal cell line CNE2 with IC_50_ value of 1*μ*M; so, further mechanistic studies should be conducted in future because nasopharyngeal carcinomas are one of the most prevalent cancers in Asia. This review also elucidates the potential role of Formononetin for the cure of several other diseases. Reported studies acclaim multiple biological properties of Formononetin but further experimentations on mechanism of its action, medicinal chemistry, and preclinical researches are yet necessary to figure out full array of its biological and pharmacological potential.

## Figures and Tables

**Table 1:** List of plants containing Formononetin and its biological activities.

Plants name		Part used/Extract	Yield of Formononetin	Functions	References
Botanical name	Common name				
*Astragalus mongholicus*	Milk vetch	Roots	10 mg /200 mg of crude extract % yield = 5 %	Anti-tumor, Anti-oxidant, Antiviral, Anti-proliferative	[25–27]

*Astragalus membranaceus*	Goat's horn	Flowers	10 mg /200 mg of crude extract % yield = 5 %	Anti-tumor, Anti-oxidant, Antiviral, Anti-proliferative	[25–27]

*Dalbergia ecastaphyllum*	Brazilian red propolis	-* *-	44.14 *μ*g/ mg (Acetate fraction) % yield = 4.414 %	Anti-tumor	[23, 28]

*Trifolium pratense*	Red clover	Above ground parts, flower heads	0.21–0.59 % (Above ground parts) 0.047–0.12% (Flower heads)	Anti-proliferative	[23, 29]

*Ononis spinosa L.*	Spiny rest harrow	Root	113.622mg/ 100 g dry plant extract % yield = 0.133 %	Beneficial for urinary and bladder infection	[9, 30]

*Glycine max*	Soya bean	-* *-	-* *-	Antioxidant, Anti-inflammatory, Anti-microbial	[15]

*Radix astragalus*	Yellow leader	Roots	0.191 *μ*g/mg % yield = 0.019 %	Osteogenic activity, Prevent postmenopausal osteoporosis, Anti-oxidant	[16, 31]

*Glycyrrhiza glabra*	Mulethi	Roots	27.856mg/ 100g % yield = 0.027 %	Anti-viral, Hepatoprotective	[30]

*Glycyrrhiza echinata*	Chinese licorice	Roots	5.218 mg/ 100 g % yield = 0.005 %	-* *-	[30]

*Cicer arietinum*	Chickpea	Seeds	14.2 mg/ 150mg % yield = 9.46 %	-* *-	[20]

*Sophora flavescens*	Shrubby	Roots	5mg/ 628g ether-soluble fraction % yield = 0.00079 %	Immuno-enhancement effects	[17, 32]
*Pycnanthus angolensis*	African nutmeg	Bark	16.2mg/180 g (n-hexane-EtOAc fraction) % yield = 0.009 %	Apoptosis inducer	[18, 33]

*Spatholobus suberectus*	Millettia	Stem	87.5 mg/25.5 g crude extract % yield = 0.343 %	Proteasome inhibitory activity	[19]

*Actaea racemosa*	Black cohosh	Rhizome	-* *-	-* *-	[22]

**Table 2:** Molecular targets of Formononetin in different types of cancer.

Cancer types	Cell lines	Treatment time	IC_50_	Molecular targets	Cell cycle arrest	References
Ovarian	ES2,OV90	48 h	40 *μ*M	p38↑, ERK1/2↓, P90RSK↓, AKT↓, P70S6K↓, ROS↑	G0/G1	[10]
	SKOV3	24 h	283.5 *μ*M	caspase3/9↑, Bax/Bcl2↑, MMP-2⊥, MMP-9⊥, p-ERK↓	-* *-	[34]
		48 h	209.3 *μ*M			
	A2780	24 h	310.0 *μ*M			
		48 h	186.1*μ*M			

Breast	MDA-MB-231, MCF-7, SK-BR-3	24 h	50 *μ*g/ml	p-GS3K*β*⊥, p-PI3K⊥, p-mTOR↑, p-AKT⊥	-* *-	[35]
	MCF-7 WS8	48 h	10 *μ*g/ml	CYP1A1↓, P450 1A1↑	-* *-	[36]
	MCF-7	24, 48h	50 *μ*M	ER*α*↑, miR-375↑, Bcl-2↓, p-AKT↑	-* *-	[37]
	4T1, MDA-MB-231	24 h	160 *μ*M	TIMP-2↑, TIMP-1↑, MMP-9↓, MMP-2↓	-* *-	[38]
	MDA-231, MDA-435	24, 48, 72 h	100 *μ*M	ER*β*↑, IGF-1R↓, PARP-1^act^, miR-375↓	-* *-	[39]
	MCF-7	24, 48, 72 h	100 *μ*M	GF1/IGF1R-PI3K/ AKT^inact^, cyclin D1↓, Bax/Bcl-2↑, Ras-p38MAPK^act^,	-* *-	[40]

Colon	SW1116,HCT116	24 h	200 *μ*M	cyclin D1↓, (MMP)2⊥, MMP9⊥, (miR)149↑, EphB3↓, PI3K/AKT⊥, STAT3⊥	G0/G1	[41]

Hepatoma	HuH-7	24 h	20 *μ*M	-* *-	-* *-	[18]

Cervical	HeLa, SiHa, CaSKi	24 h	25 *Μ*m	ROS↓, JNK⊥, caspase-8^act^, caspase-3^act^, Bax/Bcl2↑, PI3K/AKT⊥	-* *-	[42]

Laryngeal	Hep-2	24, 48 h	50 and 75 *μ*g/ml	ROS↓, CdCl2⊥	G0/G1	[23]

Lung	A549	48 h	60 mg/ml	p-Smad 2/3↓, TGF-*β*1⊥, EMT⊥	-* *-	[43]
	A549, NCI-H23	24 h	100 *μ*M	p53↑, p21↑, cyclin A↓, cyclin D1↓,	G1	[44]

Bladder	T24	24 h	200 *μ*M	T24⊥, PTEN↑, p- AKT ↓, miR-21^↓^	-* *-	[45]

Gastric	MGC-803	-* *-	-* *-	-* *-	-* *-	[46]

Esophagus	EC-109	-* *-	-* *-	-* *-	-* *-	[46]

Prostate	PC3	48 h	1.97 *μ*M	p-ERK↓, p-JNK↓, c-Myc↓ p-p38↓, cyclin B1↓, cyclin A↓, cyclin D1↓, CDK4↓, Axin↑, *β*-catenin↓, TCF-4↓	G1	[46]
	DU145, PC3	48 h	200 *μ*M	Bcl-2↓, RASD1↑, Bax↑, IGF-1 R↓	-* *-	[47]
	PC3	48 h	>12.5 *μ*M			[48]
	PC-3, DU145	48 h	80*μ*M	AKT /cyclin D1/CDK4↓	G0/G1	[49]

Nasopharyngeal	CNE2	24 h	1 *μ*M	Bax↓, bcl-2↑, p-ERK1/2↑	-* *-	[50]

Adrenal medulla	PC12	24 h	20 *μ*g/ml	ROS↑, MDA↓, GSH↓	-* *-	[51]

Multiple myeloma	U266, RPMI8226	48 h	100 *μ*M	STAT3↓, STAT5↓, JAK1↓, JAK2↓, cSrc↓, ROS↑, caspase-3^act^, PARP^clevage^	-* *-	[52]

Osteosarcoma	U2OS	48 h	80 *μ*M	Bax↑, Bcl_2_↓, miR-375↓, caspase-3↑, ERK^inact^, AKT^inact^	-* *-	[53, 54]

Glioma	U87MG, U251MG, T98G	24,48 h	100 *μ*M	E-cadherin**↓**, HDAC5↑	-* *-	[54]

Downregulation↓, Upregulation ↑, Activation  ^Act^, Inhibition⊥, Bax: Bcl-2 associated x protein, Bcl-2: B-cell lymphoma, JNK: c-Jun-N-terminal kinase, MAPK: mitogen activated protein kinase, mTOR: mammalian target of rapamycin, Cdks: cyclin dependent kinases, EKR: extracellular signal-regulated kinase.
